# Two-Stage Screening of *Metschnikowia* spp. Bioprotective Properties: From Grape Juice to Fermented Must by *Saccharomyces cerevisiae*

**DOI:** 10.3390/microorganisms12081659

**Published:** 2024-08-13

**Authors:** Julie Aragno, Pascale Fernandez-Valle, Angèle Thiriet, Cécile Grondin, Jean-Luc Legras, Carole Camarasa, Audrey Bloem

**Affiliations:** 1 UMR SPO, INRAE, Institut Agro, Université Montpellier, 34060 Montpellier, France; julie.aragno@inrae.fr (J.A.); cecile.grondin@inrae.fr (C.G.); jean-luc.legras@inrae.fr (J.-L.L.); carole.camarasa@inrae.fr (C.C.); 2 Microbial Research Infrastructure, 4710-057 Braga, Portugal

**Keywords:** bioprotection, yeast, *Metschnikowia*, wine, fermentation, *Gluconobacter*, *Brettanomyces*, aroma

## Abstract

*Gluconobacter oxydans* (Go) and *Brettanomyces bruxellensis* (Bb) are detrimental micro-organisms compromising wine quality through the production of acetic acid and undesirable aromas. Non-*Saccharomyces* yeasts, like *Metschnikowia* species, offer a bioprotective approach to control spoilage micro-organisms growth. Antagonist effects of forty-six *Metschnikowia* strains in a co-culture with *Go* or *Bb* in commercial grape juice were assessed. Three profiles were observed against Go: no effect, complete growth inhibition, and intermediate bioprotection. In contrast, *Metschnikowia* strains exhibited two profiles against Bb: no effect and moderate inhibition. These findings indicate a stronger antagonistic capacity against Go compared to Bb. Four promising *Metschnikowia* strains were selected and their bioprotective impact was investigated at lower temperatures in Chardonnay must. The antagonistic effect against Go was stronger at 16 °C compared to 20 °C, while no significant impact on Bb growth was observed. The bioprotection impact on *Saccharomyces cerevisiae* fermentation has been assessed. *Metschnikowia* strains’ presence did not affect the fermentation time, but lowered the fermentation rate of *S. cerevisiae*. An analysis of central carbon metabolism and volatile organic compounds revealed a strain-dependent enhancement in the production of metabolites, including glycerol, acetate esters, medium-chain fatty acids, and ethyl esters. These findings suggest *Metschnikowia* species’ potential for bioprotection in winemaking and wine quality through targeted strain selection.

## 1. Introduction

During winemaking, the complex ecosystem of micro-organisms plays a crucial role in fermentation, flavor profile development, and overall wine quality. The grape surface and must harbor a diverse microbial community, including yeasts (dominated by non-*Saccharomyces* species with a lower abundance of *Saccharomyces*), lactic acid bacteria (Gram-positive), and spoilage micro-organisms such as acetic acid bacteria (Gram-negative, leading to vinegar spoilage) and molds (producers of mycotoxins and off-aromas). Early in alcoholic fermentation (AF), non-*Saccharomyces* yeasts outcompete bacteria due to their superior adaptation to the must environment. However, as the ethanol concentration increases, *Saccharomyces cerevisiae* becomes the dominant species. During and after AF, certain lactic acid bacteria, acetic acid bacteria, and *Brettanomyces bruxellensis* can persist, leading to malolactic fermentation, acetic souring, and horse aroma development, respectively. Notably, *B. bruxellensis* is able to survive in stressful conditions encountered during aging and bottling (high ethanol, low oxygen, and sulfite resistance), allowing it to develop alongside bacteria [1,2,3,4]. 

Sulfur dioxide (SO_2_) is widely employed in winemaking as an antimicrobial and antioxidant agent to maintain wine quality [5,6,7,8,9,10,11,12,13,14,15,16,17,18,19]. However, a growing consumer preference is emerging for food products with a reduced additive content [20,21]. This shift is primarily driven by the potential health effects associated with SO_2_ consumption, including intolerance reactions ranging from headaches to skin, respiratory, and gastrointestinal issues [22,23,24,25]. Additionally, SO_2_ can contribute to the formation of unpleasant aromas [25,26]. In addition, studies showed a varying species-dependent resistance to SO_2_, including *B. bruxellensis* strains [6,27,28,29,30,31]. To achieve food preservation with minimal reliance on external inputs, promising microbiological alternatives like bioprotection have emerged. It involves introducing a selected living micro-organism during the pre-fermentation stage. This micro-organism is chosen for its ability to inhibit the growth of undesirable indigenous biota without negatively impacting the sensory characteristics of the final product [32]. The criteria for selecting the antagonist have been outlined by Janisiewicz and Korsten (2002) [33].

In oenology, yeast strains of the genus *Metschnikowia* are highly studied for their bioprotection effect, notably *M. pulcherrima*, *M. fructicola*, and *M. rubicola* species. These species have recently been proposed to be merged in the *Metschnikowia pulcherrima* species [34]. Studies have revealed the inhibitory effects of *M. pulcherrima* against different yeast species (*Hanseniaspora* spp., *Pichia* spp., *Torulaspora delbrueckii*, *Saccharomycodes* spp., *Zygosaccharomyces* spp., *Kluyveromyces thermotolerans*, and *Candida* spp.) [35,36,37,38,39,40,41] and molds (*Aureobasidium* spp., *Botrytis* spp., *Penicillium* spp., *Fusarium* spp., and *Alternaria* spp.) [37,42,43,44,45,46,47,48,49,50], and fewer on *B. bruxellensis* [35,40,46,51,52] and bacteria [49,51,52,53,54,55,56]. Furthermore, the addition of *Metschnikowia* yeasts to wine fermentation can bring a positive impact on the sensorial characteristics of wines. Strains used in the sequential inoculation with *Saccharomyces cerevisiae* reduce alcohol levels in wines, with a concomitant increase in glycerol [57,58,59]. Indeed, *Metschnikowia* produces many compounds such as terpenes, higher alcohols, and esters [60], which are key contributors to fruity and floral notes in wines. Studies have shown increased levels of acetate esters like isoamyl acetate (banana fruit) and phenylethyl acetate (rose aroma) in wines co-fermented with *Metschnikowia* [38,59]. 

This paper aims to investigate the genericity of bioprotection within *Metschnikowia* genus and its efficiency under winemaking conditions with *Saccharomyces cerevisiae*. Initially, the inhibitory capacity of forty-six strains of *Metschnikowia* spp. against two undesired microbes, an acetic acid bacterium (*Gluconobacter oxydans*) or a yeast *B. bruxellensis*, was assessed in modified commercial grape juice. Subsequently, four strains exhibiting diverse inhibitory profiles were evaluated for their bioprotectant effect against *G. oxydans* and *B. bruxellensis*, in a Chardonnay must at lower temperatures. Lastly, the impact of bioprotection on spoilage micro-organism populations and the fermentation process driven by *Saccharomyces cerevisiae* was determined.

## 2. Materials and Methods

### 2.1. Strain Collection, Storage, and Pre-Cultures

The complete information of the strains used in this study, including the species, the supplier, the accession number, their geographical origin, and the substrate of isolation are specified in Table S1. Yeast strains were stored in YEPD (glucose 20 g/L, peptone 20 g/L, and yeast extract 10 g/L) and glycerol 20%. The bacterial strain was stored in commercial grape juice (250 mL/L) supplemented with yeast extract 5 g/L and Tween 80 1 mL/L (the pH was adjusted to 4.8 with the addition of NaOH 32%), and then supplemented with glycerol 20%. Cells were stored at −70 °C.

Pre-cultures of *Metschnikowia* spp. and *S. cerevisiae* were carried out in liquid YEPD. The acetic acid bacteria, *G. oxydans*, and yeast *B. bruxellensis* were grown in liquid modified commercial grape juice at pH 4.8 and adjusted at pH 3.3 with HCl 37%, respectively. Pre-cultures were incubated at 28 °C with agitation for 24 h for the *Metschnikowia* species and *G. oxydans*, while, for *B. bruxellensis*, they were incubated for 48 h for bioprotection screening assays. A pre-culture of *B. bruxellensis* was grown in filtered Chardonnay must at 28 °C for 72 h and *G. oxydans* in modified commercial grape juice at pH 4.8 for 24 h at 28 °C, while *Metschnikowia* species and *S. cerevisiae* were grown at 28 °C for 24 h in YEPD and 2 mL of the pre-cultures were inoculated in 18 mL of fresh YEPD for another 24 h for fermentation assay in natural must.

### 2.2. Fermentation Media

For screening interaction, a liquid modified commercial grape juice pH 3.3, previously described in Section 2.1, was used.

Chardonnay must from Pech Rouge (Gruissan, France) was composed of 123 g/L of fructose and 116 g/L of glucose, and 105 mg/L of yeast assimilable nitrogen. For the temperature assay and pre-culture, the must was centrifuged at 4500 rpm for 10 min at 4 °C. It was then sequentially filtered through 0.45 µm and 0.22 µm membranes to remove any contaminants. The solution was maintained at 4 °C until the experiment. A mother solution of phytosterol (20 g/L) was prepared by dissolving 20 g/L of β-Sitosterol in a 1:1 (*v*/*v*) mixture of Tween 80 and ethanol. This solution was then diluted in ethanol to obtain a working solution of 5 g/L phytosterol. On the day of the experiment, this 5 g/L working solution was added to the natural must to achieve a final concentration of 5 mg/L phytosterol.

### 2.3. Interaction Assay Conditions

For the screening interaction experiment, an aliquot of pre-cultures was diluted 1000-fold in a phosphate-buffered saline (PBS) solution and counted using an Attune™ NxT Acoustic Flow Cytometer (Invitrogen, Carlsbad, CA, USA). To investigate the effect of bioprotection, *Metschnikowia* was inoculated at 10^6^ cell/mL with either *G. oxydans* at 10^3^ cell/mL or *B. bruxellensis* at 10^3^ cell/mL in synthetic grape juice at a pH of 3.3. The cultures were incubated at 22 °C. *G. oxydans* culture, *Metschnikowia* spp. culture, and the co-culture with *Metschnikowia* and *G. oxydans* were counted at day 0, 1, 2, and 7. Similarly, the *B. bruxellensis* culture and the co-culture with *Metschnikowia* spp. and *B. bruxellensis* were counted at days 0, 2, 5, and 8.

For the interaction in natural must at different temperatures, the bioprotective effect of four selected strains of *Metschnikowia* was studied. Aliquots of pre-cultures were diluted, counted using an Attune™ NxT Acoustic Flow Cytometer (Invitrogen, Carlsbad, CA, USA) to inoculate the cells. The final concentrations in filtered Chardonnay must co-cultures were 10^6^ cell/mL for *Metschnikowia* spp., 10^3^ cell/mL for *G. oxydans*, and 10^3^ cell/mL for *B. bruxellensis*. The co-cultures were incubated at either 20 °C or 16 °C. Cells were counted on selective media after 2 and 5 days of incubation.

### 2.4. Bioprotection and Fermentation Conditions in Natural Must

One-liter fermenters were filled with one liter of Chardonnay must from INRAe experimental unit Pech Rouge (Gruissan, France). The must was sterilized with addition of 600 mg/L of DMDC (Dimethyl dicarbonate) (VELCORIN^®^, Lanxess, Köln, Germany). After the spontaneous hydrolysis of the product in 72 h, the must is oxygenated for 1 h and a solution of phytosterol (5 g/L) was added to a final concentration of 5 mg/L. A sample was taken to quantify nitrogen. The fermenter with control condition with *S. cerevisiae* was added a solution of NUTRISTART^®^ (Laffort, Bordeaux, France) to adjust nitrogen concentration at 200 mg/L before inoculation. The assays’ conditions with *Metschnikowia* strains (10^6^ cell/mL), *G. oxydans* (10^3^ cell/mL), and *B. bruxellensis* (10^3^ cell/mL) were inoculated after pre-cultures were diluted and counted on Attune™ NxT Acoustic Flow Cytometer (Invitrogen, Carlsbad, CA, USA). The fermenters were incubated at 20 °C with agitation. After 48 h, nitrogen sources were quantified and NUTRISTART^®^ (Laffort, Bordeaux, France) solution was added to adjust the nitrogen concentration at 200 mg/L. The previous fermenters were inoculated with pre-cultures of *S. cerevisiae*, counted on Attune™ NxT Acoustic Flow Cytometer, at 10^6^ cell/mL and incubated at 18 °C. Weight loss was followed every hour, and the accumulation of carbon dioxide (CO_2_) released and its rate production were calculated. At 48 h and after 5 days of fermentation, cells were counted on selective media described in 2.5 and digital PCR. When the rate of production of CO_2_ was under 0.001 g/L/h, samples were taken to perform HPLC and GC-MS. The samples were centrifuged at 4500 rpm for 5 min at 4 °C and the supernatant was stored at −20 °C

### 2.5. Micro-Organism Counting

Liquid pre-cultures were diluted 1000-fold and counted on the Attune™ NxT Acoustic Flow Cytometer (Invitrogen) with the following parameters: FSC = 140 mV, SSC = 240 mV, and threshold = 1000. Gating for the yeast and bacterial quantification was performed on the window SSC-H vs. FSC-H.

The growth of *Metschnikowia* strains—A solid YEPD medium (glucose 20 g/L, peptone 20 g/L, agar 20 g/L, and yeast extract 10 g/L) was supplemented with 200 mg/L of chloramphenicol. Plates were stored at 4 °C. To enumerate the cells, an aliquot of the culture samples was sequentially diluted up to 10^−5^, and drop of 10 µL of each dilution was plated in triplicate to obtain between 10 and 100 colonies CFU/mL. Plates were incubated at room temperature and enumerated after 48 h.

The growth of acetic acid bacteria strains—A solid grape juice medium (grape juice 250 mL/L, agar 20 g/L, yeast extract 5 g/L, Tween 80 1 mL/L, and pH adjusted at 4.8 with NaOH 32%) was supplemented with 100 mg/L of cycloheximide. Plates were stored at 4 °C. Culture samples were sequentially diluted up to 10^−4^, and drop of 10 µL of each dilution was plated in triplicate to count between 10 and 100 CFU/mL. After 72 h at room temperature, plates were counted.

*Brettanomyces bruxellensis* detection by digital PCR (dPCR)—Due to the overlapping morphology of *B. bruxellensis* and *Metschnikowia* species on medium, dPCR was necessary to achieve specific identification and quantification of living and viable but non-culturable *B. bruxellensis* cells. The dPCR allows to quantify the number of DNA copies per mL with a small volume of sampling, with more precision, especially for rare target and without the need of a standard curve. The company I.A.G.E (Montpellier, France) performs the extraction and the quantification. The detection threshold is five copies of DNA/mL.

### 2.6. Chemical Analysis

Nitrogen quantification—Quantification of amino acids and ammonia has been performed by an enzymatic kit based on optic density. The free amino acids concentration has been determined by using the K-PANOPA kit from Megazyme, by manual assay according to the manufacturer’s instruction. The amino nitrogen group reacts with N-acetyl-L-cysteine (NAC) and o-phthaldialdehyde (OPA) to form a derivative measurable at 340 nm. Ammonia concentration has been determined by using the K-AMIAR kit from the same manufacturer, by manual assay according to manufacturer’s instructions. In this kit, the enzyme glutamate dehydrogenase (GIDH) transforms NADPH, ammonia, and 2-oxoglutarate to form NADP+, which will increase optical density at 340 nm. The assays were carried out on the Chardonnay must used for fermentation before inoculation of *S. cerevisiae*.

Quantification of metabolites from the central carbon metabolism—The samples from the fermentation were analyzed to quantify the metabolites from the central carbon metabolism such as glucose, fructose, ethanol, glycerol, succinate, and acetate, by high-performance liquid chromatography using the device Aminex HPX-87H ion exclusion column (Bio-Rad, Marnes-la-Coquette, France) composed of a column with styrene-divinylbenzene (SDVB) resin and a refracto-UV detector (Agilent Technologies, Santa Clara, CA, USA). A solution of sulfuric acid 1 N (MERCK 1.09072.100) was diluted in ultra-pure water to obtain a solution at 0.005 N. Then, 1 mL of sample was filtered using a syringe with a Whatman spartan 13/0.2 RC 0.2 µm filter and diluted at sixth in the sulfuric acid 0.005 N solution.

Organic volatile compound quantification—A double liquid–liquid extraction (DLLE) was performed as Rollero et al. (2015) [61] and samples were analyzed by gas chromatography coupled with a mass spectrometry as Tyibilika et al. (2023) [62]. Data were processed using OpenLab CDS 2 software (Agilent Technologies, Santa Clara, CA, USA).

### 2.7. Statistical Analysis

Analysis was performed in triplicate, except for the screening assays to follow the growth of *B. bruxellensis* or *G. oxydans* in pure culture, which were carried out eight or nine times, respectively. Data analysis was performed with the package XLSTAT 2022.4.1 (1383). Statistical comparisons between the growth in co-culture and monoculture were analyzed by ANOVA with a Dunnett test and the comparison between the effects of *Metschnikowia* strains were analyzed by ANOVA with Tukey test. Metabolite productions were compared by ANOVA with Dunnett and Tukey tests. Volatile compound production was analyzed and represented by principal component analysis (PCA).

## 3. Results

Thirty-nine strains of the *Metschnikowia pulcherrima* species, including two commercial strains used as control (LMD85 and LMD86), were selected and compared to seven *non-pulcherrima* strains, to assess the diversity of the bioprotective effect within the genus under oenological conditions. The antimicrobial activity was tested against two spoilage micro-organisms: *Gluconobacter oxydans* (Go), an acetic acid bacterium responsible for acetic acid production, and *Brettanomyces bruxellensis* (Bb), a yeast that produces phenolic off-flavors, which negatively affects wine’s sensorial properties. 

### 3.1. Different Profiles of Interaction between Gluconobacter Oxydans and a Collection of Metschnikowia Strains

**Figure 1:**
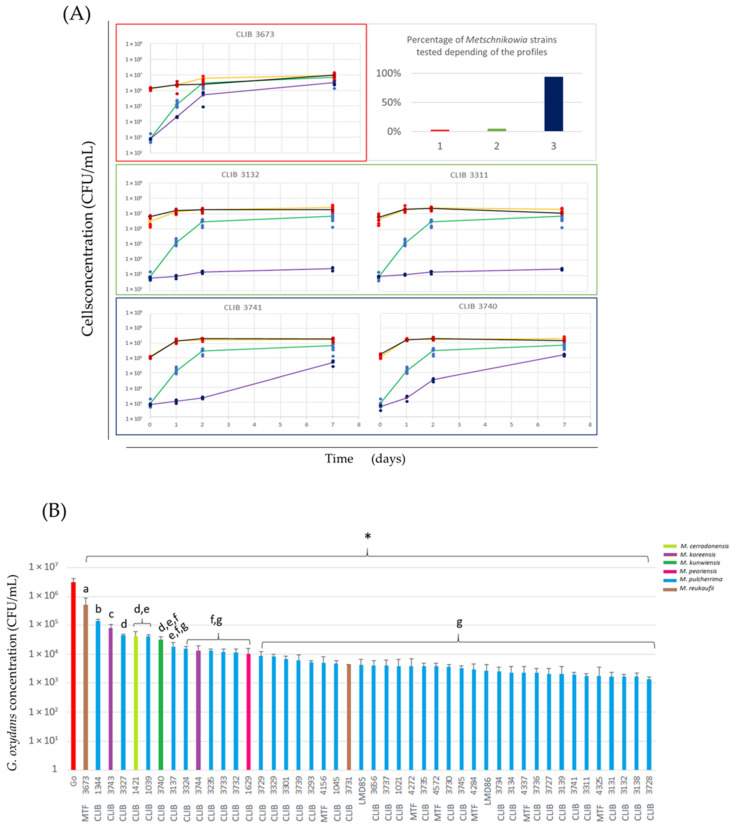
Bioprotective effect of *Metschnikowia* spp. on *Gluconobacter oxydans* (Go). (**A**) Growth profiles of *Gluconobacter oxydans* in pure culture (blue dots represent individual growth measurements from 9 replicates, and the green line shows the average growth), *Gluconobacter oxydans* in co-culture (darker blue dots represent individual growth measurements from 3 replicates, and the purple line shows the average growth of *Gluconobacter oxydans* with *Metschnikowia* spp.), *Metschnikowia* spp. in pure culture (red dots represent individual growth measurements, and the orange line shows the average growth), and *Metschnikowia* spp. in co-culture (darker red dots represent individual growth measurements from 3 replicates, and the black line shows the average growth of *Metschnikowia* spp. in co-culture). Three different profiles of bioprotection (shown in green, blue, and red frames) were identified, and the percentage of *Metschnikowia* strains tested showing these profiles are represented in barplot. (**B**) Barplot showing the population of *Gluconobacter oxydans* after two days of inoculation. (*) Significant difference between the conditions with *G. oxydans* (Dunnett test; *p*-value < 0.05); (a–g) groups according to Tukey test (*p*-value < 0.05).

The growth of the cells was monitored at inoculation, day 1, day 2, and day 7 on selective plates, after inoculation in commercial grape juice, and revealed three different inhibition profiles on bacterial growth (Figure 1A). The first profile that displays no delay in the bacterial growth when co-cultured was only observed for *Metschnikowia reukaufii* strain MTF 3673. In contrast, the second profile that shows the near-complete inhibition of *G. oxydans* growth was observed with two *Metschnikowia pulcherrima* strains CLIB 3132 and CLIB 3311. In particular, the bacterial population difference between inoculation and day 7 remains below the 1-log unit in these co-cultures. Finally, the 43 (out of 46) *Metschnikowia* remaining strains displayed a third profile (Figure S1). However, this effect is weaker than that observed with the second profile strains and disappears after 7 days. Overall, the *Metschnikowia* spp. mostly inhibit *G. oxydans* growth, but this effect depends on the strain and decreases over time. Conversely, the bacteria have no significant effect on yeast growth.

In winemaking, bioprotective strains are typically inoculated during the pre-fermentary stage. Therefore, to compare the bacterial population in interaction with each *Metschnikowia* strain, this work focused the analysis on day 2 after inoculation (Figure 1B). The concentration of *G. oxydans* was significantly higher in the pure culture (3 × 10^6^ cell/mL) compared to the co-inoculation with *Metschnikowia* strains (less than 10^6^ cell/mL). Furthermore, the bacterial population varied between co-cultures with different yeast strains. The growth of *G. oxydans* ranged from 5.2 × 10^5^ cell/mL with strain MTF 3673 (*M. reukaufii*) to 1.3 × 10^3^ cell/mL with strain CLIB 3728 (*M. pulcherrima*), highlighting the diversity and strain-dependent effects on *G. oxydans* inhibition.

### 3.2. Metschnikowia Species Bioprotective Effect against Brettanomyces Bruxellensis

**Figure 2:**
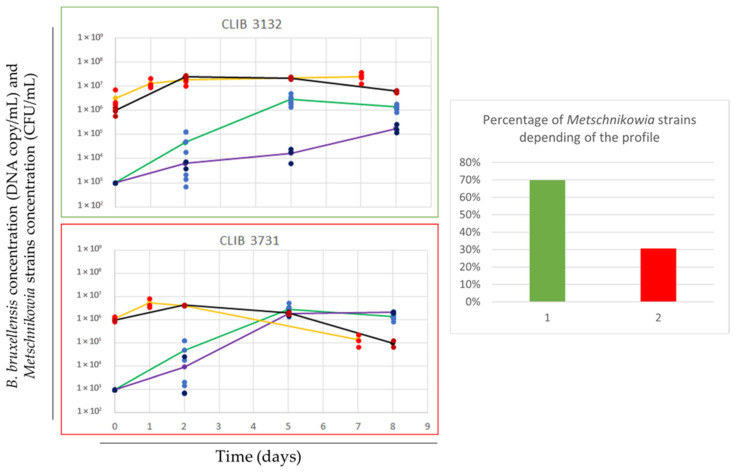
Profiles of bioprotective effect of *Metschnikowia* spp. on *B. bruxellensis* (Bb). Growth profiles of *B. bruxellensis* in pure culture (blue dots represent individual growth measurements from 8 replicates, and the green line shows the average growth), *B. bruxellensis* in co-culture (darker blue dot represent individual growth measurements from 3 replicates and the purple line shows the average growth of *B. bruxellensis* with *Metschnikowia* spp.), *Metschnikowia* spp. in pure culture (red dots represent individual growth measurements from 3 replicates, and the orange line shows the average growth), and *Metschnikowia* spp. in co-culture (darker red dots represent individual growth measurements, and the black line shows the average growth of *Metschnikowia* spp. in co-culture). Two different profiles of bioprotection (shown in green and red frame) were identified, and the percentage of *Metschnikowia* strains tested showing these profiles are represented in barplot.

Cell counts were performed using selective media (*Metschnikowia* spp.) and dPCR (*B. bruxellensis*) to investigate the interaction between the two species at 0, 2, 5, and 8 days after inoculation in commercial grape juice. The results revealed two distinct behaviors exhibited by *Metschnikowia* spp. (Figure 2). The first behavior, observed in 31 out of 46 strains tested (Figure S2), showed a delay in *B. bruxellensis* growth, especially at day 5 of co-culture. Compared to the pure culture, the decrease in the spoilage micro-organism ranged from 1 to 2-log units, as exemplified by the strains CLIB 3132 and CLIB 3736 (*M. pulcherrima*), respectively. However, while most *Metschnikowia* spp. strains delay *B. bruxellensis* growth at day 5, this effect disappears by day 8, indicating a time-dependent aspect. The second behavior, observed for 15 *Metschnikowia* spp. strains (Figure S3), displayed no significant impact on *B. bruxellensis* growth, with a difference of less than 1-log compared to the pure culture. These results support a strain-dependent bioprotective effect. Conversely, *B. bruxellensis* did not significantly impact the growth of *Metschnikowia* spp.

The analysis of *B. bruxellensis* at day 2 of co-inoculation revealed a high variability in the counted population in pure culture, ranging from 10^3^ cell/mL to 10^5^ cell/mL. Consequently, comparing the effect of bioprotection between *Metschnikowia* spp., as previously carried out in Figure 1, was not possible on day 2.

### 3.3. Effect of Temperature on Bioprotection against Spoilage Micro-Organism in Natural Must

To validate the bioprotective behavior observed in commercial grape juice, four *M. pulcherrima* strains were selected based on their diverse bioprotective profiles for further study in natural grape must. Strain CLIB 3741 was selected for its strong inhibitory activity against both acetic acid bacteria and yeast. The strains CLIB 1344 and CLIB 3138 displayed a low and an intermediate level of bioprotection, respectively. The commercial strain LMD86 was also included as control. These strains were inoculated into Chardonnay grape juice containing both *G. oxydans* (10^3^ cell/mL) and *B. bruxellensis* (10^3^ cell/mL), at either 16 °C or 20 °C. Cell populations were monitored using selective media and dPCR at day 0, 2, 5, and 8 of co-culture.

**Figure 3:**
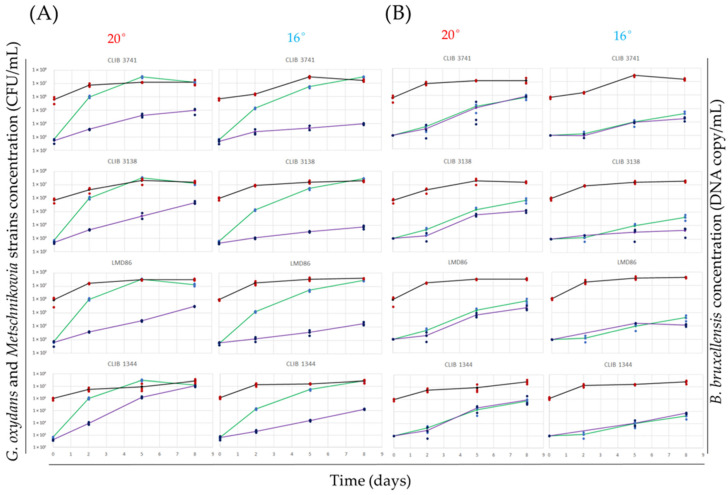
Bioprotective effect in natural must of *Metschnikowia* spp. at 20 °C and 16 °C. (**A**) Growth profile of *G. oxydans* in pure culture (blue dots represent individual growth measurements from 3 replicates, and the green line shows the average growth), *G. oxydans* in co-culture (darker blue dots represent individual growth measurements from 3 replicates, and the purple line the average growth of *G. oxydans*), and *Metschnikowia* spp. in co-culture with *G. oxydans* (red dots represent individual growth measurements from 3 replicates, and the black line the average growth of *Metschnikowia* spp.). (**B**) Growth profile of *B. bruxellensis* in pure culture (blue dots represent individual growth measurements from 3 replicates, and the green line shows the average growth), *B. bruxellensis* in co-culture with *Metschnikowia* spp. (darker blue dots represent individual growth measurements from 3 replicates, and the purple line the average growth of *B. bruxellensis*), and *Metschnikowia* spp. in co-culture with *B. bruxellensis* (red dots represent individual growth measurements from 3 replicates, and the black line the average growth of *Metschnikowia* spp.).

All four *Metschnikowia* strains exhibited bioprotective effects against acetic acid bacteria in Chardonnay must at both 16 °C and 20 °C (Figure 3A). Notably, on day 2 of the co-culture, regardless of the temperature, *Metschnikowia* strains delayed *G. oxydans* growth by 2-log units. Strain CLIB 3741 maintained a strong bioprotective effect at 20 °C after 8 days, with a significant 2-log difference compared to the pure *G. oxydans* culture. Strains CLIB 3138 and LMD86 showed a reduced antimicrobial effect with a significant 1-log difference compared to the pure culture after 8 days. Strain 1344 displayed no effect at 20 °C after 8 days, despite some inhibitory effect observed at days 2 and 5. Interestingly, the inhibitory effect on bacterial growth was significantly stronger at 16 °C compared to 20 °C. On day 8, strain CLIB 3741 inhibited bacterial growth by 3-log units at 16 °C compared to 2 log units at 20 °C. Strains CLIB 3138 and LMD86 showed a 3-log delay at 16 °C versus a 1-log difference at 20 °C. Finally, strain CLIB 1344, which showed no effect at 20 °C, reached a 2-log delay at 16 °C. At 20 °C, no significant difference was observed between the growth of *B. bruxellensis* in the pure culture or in the co-culture with *Metschnikowia* spp. strains at days 2, 5, and 8 (Figure 3B). At 16 °C, *B. bruxellensis* was not detected on day 2 of co-culture. Only on day 8 of co-culture, a slight decrease in the growth of *B. bruxellensis* was observed with strain CLIB 3138 compared to the pure culture. The three other strains, CLIB 3741, LMD86, and CLIB 1344, showed no difference in the *B. bruxellensis* population. Overall, the bioprotective effect of *Metschnikowia* spp. is dependent on both the strain used and the fermentation temperature. Interestingly, the antimicrobial properties were found to be more effective at lower temperatures, particularly against acetic acid bacteria compared to *B. bruxellensis*.

### 3.4. Influence of Bioprotection on the Fermentation Process by Saccharomyces cerevisiae

This study aims to evaluate the impact of a bioprotection practice using four strains of *M. pulcherrima* on the progress of wine fermentation and the metabolic performances of *S. cerevisiae*. The strains of *M. pulcherrima*, *G. oxydans*, and *B. bruxellensis* are inoculated in Chardonnay must at 20 °C for 48 h to assess the bioprotection. Subsequently, an *S. cerevisiae* strain was inoculated, and the nitrogen content was adjusted to ensure fermentation by *S. cerevisiae* at 18 °C. The main fermentation parameters were then compared between the different modalities, including: the maximal rate of CO_2_ production (Rmax) of the *Metschnikowia* and *S. cerevisiae* strains, the CO_2_ production before inoculating *S. cerevisiae* (at 48 h), the time required for complete fermentation, the Rmax at 60% of fermentation, and the final CO_2_ concentration. To analyze the yeast metabolism, the formation of compounds from central carbon metabolism and volatile compounds were quantified at the end of fermentation. In parallel, the spoilage micro-organism populations and *Metschnikowia* strain populations were quantified on days 2 and 5 after inoculation using dilution plating on selective media and digital PCR (dPCR).

First, the Rmax and the CO_2_ produced at 48 h by *Metschnikowia* strains were investigated (Table 1A). Strain CLIB 3741 exhibited the highest Rmax (0.17 g/L/h), followed by the strains LMD86 and CLIB 3138 (0.15 g/L/h and 0.13 g/L/h, respectively). The strain CLIB 1344 had the lowest Rmax (0.10 g/L/h). The CO_2_ production before the inoculation of *S. cerevisiae* also varied between strains. Strains CLIB 3741, CLIB 3138, and LMD86 produced between 3.4 g/L and 3.9 g/L of CO_2_, while strain CLIB 1344 produced only 1.2 g/L of CO_2_. This reflects the lower fermentation efficiency of CLIB 1344 compared to the three other strains.

When co-cultured with the spoilage micro-organisms (*G. oxydans* and *B. bruxellensis*), *S. cerevisiae* showed no significant difference in final CO_2_ concentration, and Rmax or the Rmax at 60% of fermentation (Table 1A). However, the fermentation time is reduced by 41 h in the presence of acetic acid bacteria and *B. bruxellensis* compared to *S. cerevisiae* alone. This reduction corresponds to the time spent fermenting before *S. cerevisiae* inoculation, suggesting the spoilage micro-organisms do not impact the fermentation capacities of *S. cerevisiae*. 

Conversely, the presence of *Metschnikowia* strains appeared to slow down *S. cerevisiae* fermentation. While the Rmax with spoilage micro-organisms was 0.83 g/L/h, this rate decreased from 0.58 g/L/h to 0.65 g/L/h in the co-fermentation with *Metschnikowia* strains. Additionally, the fermentation time, calculated from the addition of *S. cerevisiae*, significantly decreased by 14 h compared to *S. cerevisiae* alone, potentially due to some sugar consumption of the *Metschnikowia* strains. However, the Rmax at 60% of fermentation and the final CO_2_ production remained unaffected by the presence or absence of *Metschnikowia* strains. These results suggest that *G. oxydans* and *B. bruxellensis* do not affect *S. cerevisiae* fermentation. However, the presence of *Metschnikowia* may slow down *S. cerevisiae* fermentation by decreasing the Rmax. In addition, *Metschnikowia* strains display a strain-dependent ability to ferment sugars.

Regarding the formation of central carbon metabolites (ethanol, glycerol, succinate, and acetate), no significant difference was observed between the fermentation with *S. cerevisiae* alone and *S. cerevisiae* co-fermented with spoilage micro-organisms (Table 1B). When *Metschnikowia* strains were inoculated 48 h before *S. cerevisiae* and spoilage micro-organisms, the production of ethanol, succinate, and acetate remained similar. However, a variation in glycerol production was observed. Compared with the final glycerol concentration measured with *G. oxydans*, *B. bruxellensis*, and *S. cerevisiae* fermentation (6.75 g/L), the co-fermentation of these micro-organisms with the strains LMD86, CLIB 1344, or CLIB 3138 resulted in an increase in glycerol production by approximately 1.5 g/L (ranging from 8.2/L to 8.5 g/L). Interestingly, strain CLIB 3741 produced significantly more glycerol (9.2 g/L) while maintaining similar levels of ethanol, succinate, and acetate.

**Figure 4:**
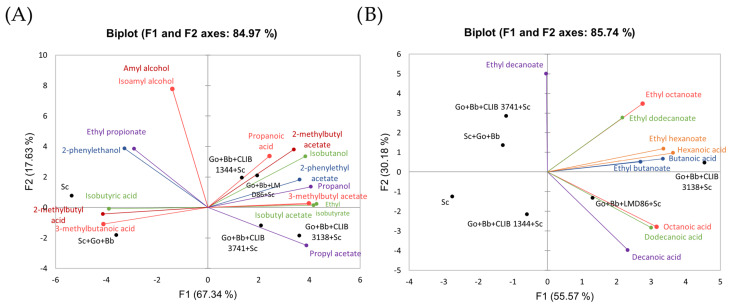
Principal component analysis of the volatile compounds produced by *Metschnikowia* strains and *S. cerevisiae* during fermentation. (**A**) Higher alcohols and esters (in red, the compounds derived from leucine; in darker red, compounds derived from isoleucine; in green, the compounds derived from valine; in blue, the compounds derived from phenylalanine; and, in purple, the compounds derived from threonine). (**B**) Medium-chain fatty acids and their derived ethyl esters represented with one color per group.

To gain insights into the impact of the use of *Metschnikowia* strains as a bioprotective agent on the formation of volatile compounds, two principal component analyses (PCAs) were performed (Figure 4) to compare either the formation of volatile compounds derived from the Ehrlich pathway, which is linked to yeast nitrogen and carbon metabolism, or medium-chain fatty acids and their corresponding ethyl esters, which are associated with lipid metabolism via acetyl-CoA. Each analysis explained 85% of the variability of the studied dataset. It becomes clear that the fermentations for which a bioprotection treatment was applied differed distinctly from the control fermentations (*S. cerevisiae* alone or in presence of spoilage micro-organisms) in their production of Ehrlich volatile compounds, regardless of the specific *Metschnikowia* strain used (Figure 4A). The volatile profiles of the wines obtained with bioprotection exhibited a higher abundance of acetate esters, specifically amyl acetate, 2-phenylethyl acetate, isoamyl acetate, isobutyl acetate, and propyl acetate, and some higher alcohols (propanol and isobutanol) (Table S2). Conversely, fermentations with *S. cerevisiae* alone or in the presence of *G. oxydans* and *B. bruxellensis* produced more branched acids (isobutyric acid, valeric acid, and isovaleric acid) and 2-phenylethanol (Table S3). Regarding the formation of MCFAs and their ethyl ester derivatives (Figure 4B), fermentations with bioprotection using strains LMD86 and, especially, *M. pulcherrima* CLIB 3138 stood out with increased productions. Applying bioprotection treatments with *Metschnikowia* strains CLIB 1344 and CLIB 3741 did not alter the final production of MCFAs and their ethyl esters compared to the control fermentations (*S. cerevisiae* alone, and *S. cerevisiae* with spoilage micro-organisms) (Table S3).

In addition, the population of spoilage micro-organisms was measured on days 2 and 5 of fermentation using selective media and dPCR. While no antimicrobial effect against *B. bruxellensis* was observed, a significant decrease in 3-log units in the growth of *G. oxydans* was detected in the co-culture with *Metschnikowia* strains (Figure S4). These results suggest that the bioprotection capacities of *Metschnikowia* are still present even in the presence of *S. cerevisiae*, under oenological conditions.

## 4. Discussion

In recent years, bioprotection has gained attention as an alternative to sulfur dioxide utilization in winemaking. *Metschnikowia* is a well-studied genera due to its antimicrobial activity, particularly within the *Metschnikowia pulcherrima* clade [34]. Numerous studies demonstrate the antagonist effect of these yeasts against fungal pathogens [37,39,41,63,64]. However, research on their efficacy against *Brettanomyces bruxellensis* and bacteria, especially on acetic acid bacteria, remains limited. This study aims to assess the genericity of bioprotection effects within the genus *Metschnikowia*, including some non-pulcherrima strains, against these two major spoilage micro-organisms *B. bruxellensis* and *G. oxydans*, in both synthetic (modified commercial grape juice) and natural must, as well as its impact on fermentation by *S. cerevisiae*.

This work revealed the significant variability in bioprotective properties against the wine spoilage bacterium *Gluconobacter oxydans* within a collection of 46 *Metschnikowia* spp. strains from different species, geographical origins, and substrates. By characterizing the growth dynamics of the bacterium, three bioprotection profiles were identified for the first time: normal bacterial growth, no bacterial growth, or delayed bacterial growth in the presence of the bioprotective agent. The latter behavior is the most common, suggesting that the yeast exerts a bacteriostatic effect on *G. oxydans*, preventing an efficient growth. Most studies involving commercial strains only tested a mix or one strain of *M. pulcherrima* to assess the bioprotection effect against wine-bacteria [51,52,55,65]. In addition, screening studies with several *M. pulcherrima* strains, from 2 to 10 strains tested, against *Oenococcus oeni*, *Lactobacillus* sp., and *Pediococcus* sp., were mainly carried out in double-layer agar plates. The inhibition halos were measured on one specific day and the results highlighted a strain-dependent bioprotective capacity [49,54,65]. However, no growth kinetics were monitored.

Similarly, the bioprotective effect against the spoilage yeast *B. bruxellensis* depends on the *Mestschnikowia* strain used, with two observed behaviors: a total lack of inhibition (30% of the 46 strains tested) or the induction of delayed growth of *B. bruxellensis* (70% of *Metschnikowia* isolates). These observations are supported by the literature data, which report either an absence of bioprotective effect of seven *M. pulcherrima* grown in the presence of seven isolates of *Dekkera bruxellensis* [66] or an antimicrobial effect of varying intensity depending on the strain (from 5 to 15 strains of *M. pulcherrima* tested) [35,40,67]. 

It is noteworthy that only 4 (*M. pulcherrima* CLIB 3132, *M. pulcherrima* CLIB 3131, *M. pulcherrima* MTF 4325, and *M. pulcherrima* CLIB 3139) of the 10 strains with the strongest bioprotective effect against acetic acid bacteria were those with the most significant antimicrobial effect on *B. bruxellensis*. On the contrary, some strains, such as *M. pulcherrima* CLIB 3138, displayed a good bioprotective efficacy against *G. oxydans* but did not alter the growth of *B. bruxellensis*. This indicates that the bioprotective effect depends on both the bioprotective strain and the target contaminating micro-organism. Furthermore, the antimicrobial effect of *Metschnikowia* strains against *B. bruxellensis* observed on a synthetic medium (modified commercial grape juice) is significantly reduced on a natural Chardonnay must. Regarding *G. oxydans*, the *Metschnikowia* strain bioprotective effect, conserved on Chardonnay grape juice, is more pronounced at 16 °C compared to 20 °C. This could be related to the very weak activity of acetic acid bacteria at this temperature [68], as reported in the literature, while *Metschnikowia* yeasts are still capable of growing efficiently [69]. Limited information is available regarding bioprotection mechanisms, which can be categorized into passive (e.g., competition for oxygen, nitrogen, and iron) and active mechanisms (e.g., production of killer toxins, pulcherriminic acid, enzymatic activities, and quorum sensing) as summarized in Puyo et al. (2023) [70]. Overall, all these observations should be considered in practice when implementing microbial bioprotection strategies in pre-fermentation treatments.

Bioprotection treatment involves the use of micro-organisms, whose growth and metabolic activity can lead to changes in both the fermentation performance of the yeast strain used to conduct fermentation—in general, *S. cerevisiae*—and the final composition of wines [71]. It is, therefore, imperative to establish that this pre-treatment does not alter either the course of fermentation or the sensory quality of the wines. 

The sequential inoculation of *M. pulcherrima* highlighted the negative consequence on *S. cerevisiae* fermentation capacities. A 25% decrease in the Rmax but no impact on the rate at 60% of the fermentation of *S. cerevisiae* were observed in the presence of *M. pulcherrima*, showing an effect at the beginning of fermentation. Furthermore, the impact was less pronounced compared to the data reported in the literature by Seguinot et al. (2020) [72], which could be explained in our study by the addition of nutrients at the same time as the *S. cerevisiae* inoculation. This aligns with Barbosa et al. (2018) [73], who also observed a lower capacity of fermentation and a decrease in the *S. cerevisiae* maximum population (3.5-fold) in this condition with the addition of nitrogen sources, limiting the competition between the species. They hypothesize a competition for other nutrients or the production of inhibitory metabolites by *M. pulcherrima.* Additions in nutrients in this work consequently limit the impact on the final concentration of CO_2_ and the fermentation duration. The decrease in duration could be attributed to the consumption of sugar (10 g/L) by *Metschnikowia* strains before *S. cerevisiae* inoculation.

Regarding the production of the central metabolites ethanol, succinate, and acetate, responsible for a burning sensation, bitter-salty taste, and sour aroma in high concentrations, the bioprotection treatment using *M. pulcherrima* yeasts did not modify their final concentration in the wines. This result is surprising, as many studies report a decrease in the formation of ethanol and acetate during mixed *S. cerevisiae*/*M. pulcherrima* fermentations [57,58,59,73,74,75,76]. This could be related to differences in the implementation of the cultures, particularly the use of lower temperatures, which limit the growth and activity of *M. pulcherrima* yeasts. The only notable difference related to the bioprotection treatment is a substantial increase in glycerol formation, which leads to a fullness and sweetness to the wine, regardless of the *M. pulcherrima* strain. This results, on the one hand, from the high capacity of the *M. pulcherrima* species to produce glycerol [62,77,78], but also from a positive interaction phenomenon between the two species, favoring the production of glycerol by *S. cerevisiae* [72]. Beyond their influence on *Saccharomyces cerevisiae*’s fermentation performance, the impact of non-*Saccharomyces* yeasts on a wine’s sensorial profile holds significant importance. This factor will ultimately determine the adoption of these yeasts by winemakers. Wines produced using a bioprotection treatment with the *Metschnikowia* strain exhibit higher concentrations of acetate esters (responsible for fruity and floral aromas), higher alcohols (isobutanol and propanol with a solvent aroma), ethyl isobutyrate (fruity aroma), and propanoic acid (apple aroma), and, conversely, lower levels of branched fatty acids (isovaleric, valeric, and isobutyric acids, related to rotten fruit, fruity, and apple rot/butter, respectively), compared to wines from *S. cerevisiae*-alone fermentations. Sensory analysis should be performed in the near future to validate the previous observed effects in our study and strengthen the interest in *Metschnikowia* spp., to complexify the sensorial profiles of wine.

To confirm the generality of these findings, a broader range of *Metschnikowia* species from diverse environments should be investigated. Additionally, post-fermentation studies are essential in order to evaluate the long-term impact of bioprotection on metabolite and aroma profiles, as well as microbial community dynamics.

## 5. Conclusions

In conclusion, this work highlights a general capacity of bioprotection by several *Metschnikowia* species with a varying effect depending on the time, the target, and the medium (commercial grape juice or Chardonnay must). Further investigation needs to be carried out in different natural must. In addition, the four strains selected for their varying bioprotective effect showed a maintained antagonistic capacity at lower temperatures. Moreover, the bioprotection did not negatively impact the fermentation by *Saccharomyces cerevisiae* and the wine composition. In contrary, glycerol, and a fruity and floral aroma production were enhanced, suggesting better sensorial properties, needing to be verified by sensory analysis. This study points out bioprotection as a promising alternative to inputs such as sulfur dioxide.

## Figures and Tables

**Table 1:** Impact of *Metschnikowia* strains used as bioprotective agents on the fermentation and metabolic performances of *S. cerevisiae* (Sc) during wine fermentation. (**A**) Table presenting the fermentation parameters of *Metschnikowia* spp. at 48 h and *S. cerevisiae* (Sc) at the end of fermentation. (**B**) Table presenting the quantification of central carbon metabolites at the end of fermentation. NA indicates not attributed. (*) Significant difference between the conditions with *S. cerevisiae* (Sc) (Dunnett test; *p*-value < 0.05); (**) Significant difference between the condition with *S. cerevisiae* (Sc), *G. oxydans* (Go), and *B. bruxellensis* (Bb) (Dunnett test; *p*-value < 0.05); (a–c) Groups according to Tukey test (*p*-value < 0.05). Absence of letters or symbol signify no differences between the conditions.

**(A)**						
**Conditions**	**Sc**	**Go+Bb+Sc**	**Go+Bb+** **3741+Sc**	**Go+Bb+** **3138+Sc**	**Go+Bb+** **Initia+Sc**	**Go+Bb+** **1344+Sc**
**Rmax *Metschnikowia* (g/L/h)**	NA	NA	0.17 ± 0.01 (a)	0.13 ± 0.01 (a,b)	0.15 ± 0.01 (b)	0.10 ± 0.01 (c)
**CO_2_ Metsch (48 h) (g/L)**	NA	NA	3.98 ± 0.44 (a)	3.45 ± 0.13 (a)	3.52 ± 0.61 (a)	1.22 ± 1.11 (b)
**Rmax Sc (g/L/h)**	0.73 ± 0.03	0.83 ± 0.1	0.58 ± 0.02 (**)	0.63 ± 0.03 (**)	0.653 ± 0.02 (**)	0.613 ± 0.02 (**)
**Rmax 60% fermentation (g/L/h)**	0.35 ± 0.01	0.42 ± 0.04	0.40 ± 0.01	0.42 ± 0.01	0.38 ± 0.02	0.41 ± 0.02
**Tf fermentation (h)**	421.18 ± 13.27	379.935 ± 0.01 (*)	411.26 ± 0.01 (**)	407.05 ± 0 (**)	407.05 ± 0 (**)	407.05 ± 0 (**)
**CO_2_ final (g/L)**	110.80 ± 0.20	110.03 ± 0.26 (*)	109.43 ± 0.37	110.98 ± 2.28	110.21 ± 0.90	108.18 ± 2.55
**(B)**						
**Conditions**	**Sc**	**Go+Bb+Sc**	**Go+Bb+** **3741+Sc**	**Go+Bb+** **3138+Sc**	**Go+Bb+** **Initia+Sc**	**Go+Bb+** **1344+Sc**
**Ethanol (g/L)**	122 ± 0.4 (a)	121 ± 0.5 (a,b)	119 ± 0.1 (c)	123 ± 0.6 (a)	121 ± 0.7 (a,b,c)	119 ± 1.5 (b,c)
**Glycerol (g/L)**	7 ± 0.01 (c)	6.8 ± 0.06 (c)	9.2 ± 0.17 (a)	8.5 ± 0.21 (b)	8.3 ± 0.19 (b)	8.2 ± 0.26 (b)
**Succinate (g/L)**	1.6 ± 0.03 (a)	1.4 ± 0.02 (a,b)	1.6 ± 0.01 (a,b)	1.5 ± 0.06 (b)	1.5 ± 0.04 (a,b)	1.6 ± 0.06 (a,b)
**Acetate (g/L)**	0.20 ± 0.01 (a)	0.16 ± 0.03 (a,b)	0.16 ± 0.02 (a,b)	0.16 ± 0.01 (a,b)	0.12 ± 0.02 (b)	0.14 ± 0.04 (b)

## Data Availability

The original contributions presented in this study are included in the article; further inquiries can be directly asked to the corresponding author.

## Supplementary Materials

The following supporting information can be downloaded at: https://www.mdpi.com/article/10.3390/microorganisms12081659/s1, Table S1: Origin and substrate of isolation of the strain studied for bioprotection properties; Figure S1: Growth kinetics of the screening assay with *Metschnikowia* strains from the profile number 3, against *G. oxydans*; Figure S2: Growth kinetics of the screening assay with *Metschnikowia* strains from the profile number 1, against *B. bruxellensis*; Figure S3: Growth kinetics of the screening assay with *Metschnikowia* strains from the profile number 2, against *B. bruxellensis*; Table S2: Production of volatile compounds derived from amino acids, at the end of fermentation depending on the conditions; Table S3: Production of volatile compounds derived from medium-chain fatty acids, at the end of fermentation depending on the conditions; Figure S4: Growth followed at day 2 and day 5, of the spoilage micro-organisms *G. oxydans* (Go) and *B. bruxellensis* and the yeast *M. pulcherrima* in Chardonnay must.
