# Supplementary material for: Two-Stage Screening of Metschnikowia spp. Bioprotective Properties: From Grape Juice to Fermented Must by Saccharomyces cerevisiae
Source: Microorganisms. 2024 Aug 13;12(8):1659. doi: 10.3390/microorganisms12081659 (PMC11356803; doi:10.3390/microorganisms12081659)
Supplement: Supplementary file 1 [file microorganisms-12-01659-s001.zip › microorganisms-3134512-SI.pdf]

Specie	Supplier	N° accession	Geographical origin	Substrate
Metschnikowia cerradonensis	INRAe (Cirm-Levures)	**CLIB 1421**	Guyane, France	Hibiscus flower
Metschnikowia koreensis	INRAe (Cirm-Levures)	**CLIB 3743**	Mayotte, France	White flower
Metschnikowia koreensis	INRAe (Cirm-Levures)	**CLIB 3744**	Réunion, France	Fermented pineapple must (3 days)
Metschnikowia kunwiensis	INRAe (Cirm-Levures)	**CLIB 3740**	Nouvelle-Aquitaine, France	Vine bark
Metschnikowia peoriensis	INRAe (Cirm-Levures)	**CLIB 1629**	Guyane, France	Flower
Metschnikowia pulcherrima	INRAe (Cirm-Levures)	**CLIB 3728**	Occitanie, France	Fermented redcurrant
Metschnikowia pulcherrima	INRAe (Cirm-Levures)	**CLIB 3131**	Occitanie, France	Must of viognier grape variety
Metschnikowia pulcherrima	INRAe (Cirm-Levures)	**CLIB 3139**	Occitanie, France	Must of sauvignon grape variety
Metschnikowia pulcherrima	INRAe (Cirm-Levures)	**CLIB 3132**	Occitanie, France	Must of viognier grape variety
Metschnikowia pulcherrima	INRAe (Cirm-Levures)	**CLIB 3134**	Occitanie, France	Must of viognier grape variety
Metschnikowia pulcherrima	INRAe (Cirm-Levures)	**CLIB 3137**	Occitanie, France	Must of sauvignon grape variety
Metschnikowia pulcherrima	INRAe (Cirm-Levures)	**CLIB 3311**	Occitanie, France	Must of viognier grape variety
Metschnikowia pulcherrima	INRAe (Cirm-Levures)	**CLIB 3730**	Aveyron, France	Orchis pyramidal (Anacamptis pyramidalis)
Metschnikowia pulcherrima	INRAe (UMR SPO)	**MTF 4325**		
Metschnikowia pulcherrima	INRAe (Cirm-Levures)	**CLIB 1021**	Bretagne, France	Filtered apple raw juice
Metschnikowia pulcherrima	INRAe (Cirm-Levures)	**CLIB 1045**	Bretagne, France	Apple raw juice
Metschnikowia pulcherrima	Lallemand	**LMD85**		
Metschnikowia pulcherrima	INRAe (Cirm-Levures)	**CLIB 3733**	Centre Val de Loire, France	Fermented cherries (3 days)
Metschnikowia pulcherrima	INRAe (Cirm-Levures)	**CLIB 3734**	Occitanie, France	Mansois grape berries (Marcillac)
Metschnikowia pulcherrima	INRAe (Cirm-Levures)	**CLIB 3739**	Nouvelle-Aquitaine, France	Fermented black grapes (2 days)
Metschnikowia pulcherrima	INRAe (UMR SPO)	**MTF 4156**	Occitanie, France	Apple
Metschnikowia pulcherrima	INRAe (UMR SPO)	**MTF 4272**	Occitanie, France	Grape juice
Metschnikowia pulcherrima	INRAe (UMR SPO)	**MTF 4337**	Occitanie, France	Organic grape juice
Metschnikowia pulcherrima	INRAe (UMR SPO)	**MTF 4284**	Occitanie, France	Fermented organic grape juice
Metschnikowia pulcherrima	INRAe (Cirm-Levures)	**CLIB 1039**	Bretagne, France	Apple raw juice
Metschnikowia pulcherrima	INRAe (Cirm-Levures)	**CLIB 1344**	Californie, United States of America	Berries of Vitis labrusca (Concord grapes)
Metschnikowia pulcherrima	INRAe (Cirm-Levures)	**CLIB 3235**	Occitanie, France	Must of sauvignon grape variety
Metschnikowia pulcherrima	INRAe (Cirm-Levures)	**CLIB 3324**	Occitanie, France	Must of viognier grape variety
Metschnikowia pulcherrima	INRAe (Cirm-Levures)	**CLIB 3327**	Occitanie, France	Must of viognier grape variety
Metschnikowia pulcherrima	INRAe (Cirm-Levures)	**CLIB 3329**	Occitanie	Must of sauvignon grape variety
Metschnikowia pulcherrima	Lallemand	**LMD86**		
Metschnikowia pulcherrima	INRAe (Cirm-Levures)	**CLIB 3729**	Aveyron, France	Orchis pyramidal (Anacamptis pyramidalis)
Metschnikowia pulcherrima	INRAe (Cirm-Levures)	**CLIB 3138**	Occitanie, France	Must of sauvignon grape variety
Metschnikowia pulcherrima	INRAe (Cirm-Levures)	**CLIB 3293**	Occitanie, France	Must of viognier grape variety
Metschnikowia pulcherrima	INRAe (Cirm-Levures)	**CLIB 3301**	Occitanie, France	Must of sauvignon grape variety
Metschnikowia pulcherrima	INRAe (Cirm-Levures)	**CLIB 3656**	Ile de France, France	Lilac flower
Metschnikowia pulcherrima	INRAe (Cirm-Levures)	**CLIB 3736**	Occitanie, France	Fermented black grape Mansois (3 days)
Metschnikowia pulcherrima	INRAe (Cirm-Levures)	**CLIB 3727**	Aveyron, France	Fermented redcurrant
Metschnikowia pulcherrima	INRAe (Cirm-Levures)	**CLIB 3741**	Nouvelle-Aquitaine, France	Fermented wild strawberries (2 days)
Metschnikowia pulcherrima	INRAe (Cirm-Levures)	**CLIB 3735**	Occitanie, France	Mansois grape berries (Marcillac)
Metschnikowia pulcherrima	INRAe (Cirm-Levures)	**CLIB 3737**	Occitanie, France	Fermented white grapes (2 days)
Metschnikowia pulcherrima	INRAe (Cirm-Levures)	**CLIB 3745**	Ile de France, France	Scleroderma sp.
Metschnikowia pulcherrima	INRAe (Cirm-Levures)	**CLIB 3732**	Centre Val de Loire, France	Fermented cherries (4 days)
Metschnikowia pulcherrima	INRAe (UMR SPO)	**MTF 4572**	Pyrénées-Orientales, France	Grape must
Metschnikowia reukaufii	INRAe (UMR SPO)	**MTF 3673**	Occitanie, France	Rubus fructicosa berry
Metschnikowia reukaufii	INRAe (Cirm-Levures)	**CLIB 3731**	Provence, France	Honeysuckle (Lonicera periclymenum)
Gluconobacter oxydans	ISVV (CRBO)	**08ba05**	Nouvelle-Aquitaine, France	Grape
Brettanomyces bruxellensis	ISVV (CRBO)	**L0516**	Nouvelle-Aquitaine, France	Wine
Saccharomyces cerevisiae	INRAe (UMR SPO)	**EC1118**		

Table S1: Origin and substrate of isolation of the strain studied for bioprotection properties.

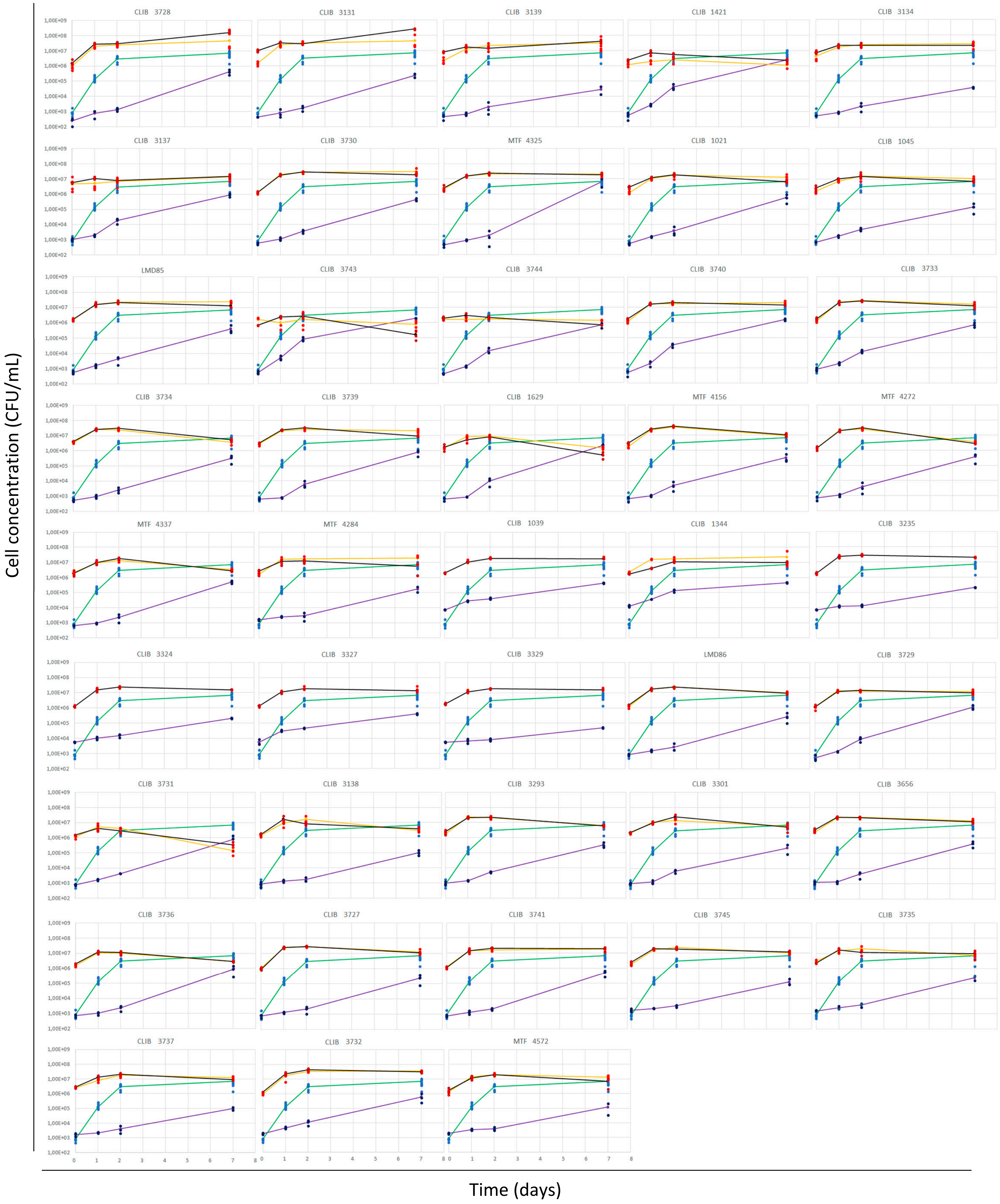

Figure S1: Growth kinetics of the screening assay with *Metschnikowia* strains from the profile number 3, against *G. oxydans*. Growth profiles of *Gluconobacter oxydans* in pure culture (blue dots represent individual growth measurements from 9 replicates, the green line shows the average growth), *Gluconobacter oxydans* in co-culture (darker blue dots represent individual growth measurements from 3 replicates and the purple line shows the average growth of Gluconobacter oxydans with *Metschnikowia* sp.), *Metschnikowia sp*. in pure culture (red dots represent individual growth measurements, the orange line shows the average growth), *Metschnikowia sp*. in co-culture (darker red dots represent individual growth measurements and the black line shows the average growth of *Metschnikowia sp*. in co-culture).

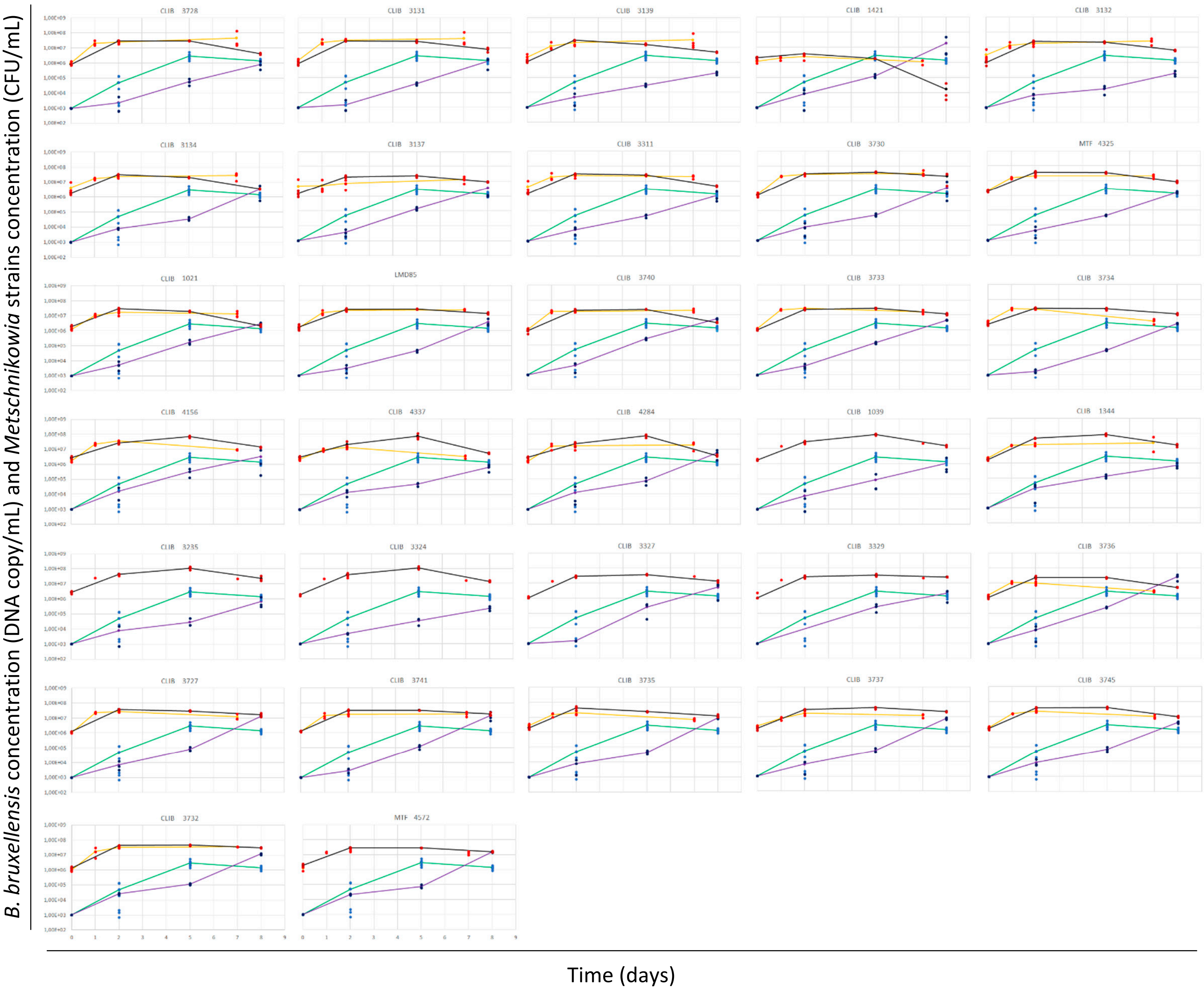

Figure S2: Growth kinetics of the screening assay with *Metschnikowia* strains from the profile number 1, against *B. bruxellensis*. Growth profiles of *B. bruxellensis* in pure culture (blue dots represent individual growth measurements from 8 replicates, the green line shows the average growth), *B. bruxellensis* in co-culture (darker blue dot represent individual growth measurements from 3 replicates and the purple line shows the average growth of *B. bruxellensis* with *Metschnikowia sp.*), *Metschnikowia sp.* in pure culture (red dots represent individual growth measurements, the orange line shows the average growth), *Metschnikowia sp.* in co-culture (darker red dots represent individual growth measurements and the black line shows the average growth of *Metschnikowia sp*. in co-culture).

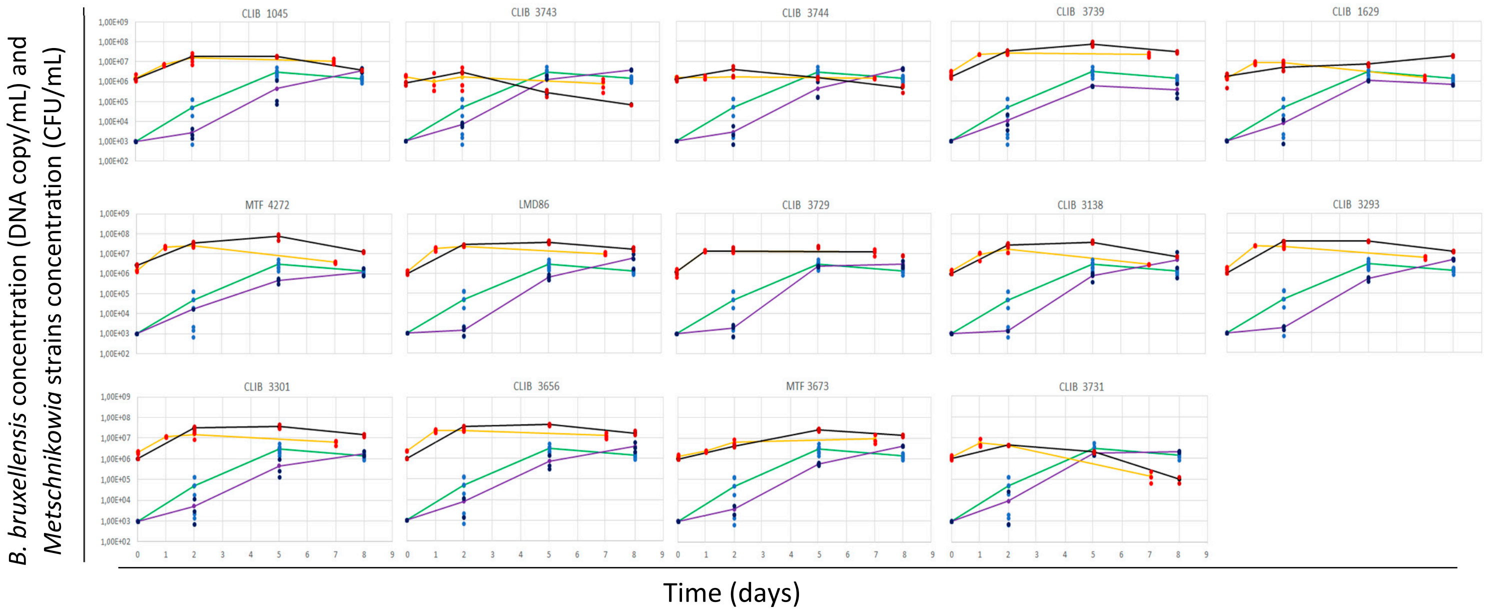

Figure S3: Growth kinetics of the screening assay with *Metschnikowia* strains from the profile number 2, against *B. bruxellensis*. Growth profiles of *B. bruxellensis* in pure culture (blue dots represent individual growth measurements from 8 replicates, the green line shows the average growth), *B. bruxellensis* in co-culture (darker blue dot represent individual growth measurements from 3 replicates and the purple line shows the average growth of *B. bruxellensis* with *Metschnikowia sp.*), *Metschnikowia sp.* in pure culture (red dots represent individual growth measurements, the orange line shows the average growth), *Metschnikowia sp.* in co-culture (darker red dots represent individual growth measurements and the black line shows the average growth of *Metschnikowia sp*. in co-culture).

Conditions	Phenylalanine: 2-phenylethanol	Phenylalanine: 2-phenylethyl acetate	Valine: Isobutanol	Valine: Isobutyl acetate	Valine: Isobutyric acid	Valine: Ethyl isobutyrate	Leucine: Isoamyl alcohol	Leucine: 3-methylbutyl acetate	Leucine: 3-methylbutanoic acid
**Sc**	70,13 ± 3,70 (a)	0,62 ± 0,09 (d)	24,67 ± 0,64 (c)	0,02 ± 0,00 (c)	1,66 ± 0,10 (a,b)	0,49 ± 0,02 (b)	295,36 ± 16,64 (a)	2,65 ± 0,13 (c)	1,88 ± 0,08 (a)
**Go+Bb+Sc**	51,57 ± 1,60 (b,c)	0,59 ± 0,09 (d)	21,00 ± 1,95 (c)	0,04 ± 0,01 (c)	1,72 ± 0,24 (a)	0,60 ± 0,05 (b)	272,16 ± 0,39 (a)	3,83 ± 0,27 (c)	1,46 ± 0,18 (b)
**Go+Bb+CLIB 3741+Sc**	38,29 ± 1,02 (d)	1,37 ± 0,27 (a)	78,52 ± 7,79 (b)	0,47 ± 0,12 (a,b)	0,91 ± 0,11 (c,d)	0,85 ± 0,03 (a)	269,17 ± 9,33 (a)	13,22 ± 2,19 (a)	0,45 ± 0,06 (c)
**Go+Bb+CLIB 3138+Sc**	41,82 ± 2,24 (d)	1,61 ± 0,10 (b)	74,80 ± 6,33 (b)	0,59 ± 0,09 (a)	0,87 ± 0,12 (d)	0,90 ± 0,04 (a)	248,53 ± 10,65 (a)	9,97 ± 0,55 (b)	0,67 ± 0,09 (c)
**Go+Bb+LMD86+Sc**	57,33 ± 1,81 (b)	2,01 ± 0,06 (b)	103,54 ± 3,09 (a)	0,53 ± 0,01 (a)	1,29 ± 0,08 (b,c)	0,84 ± 0,03 (a)	298,63 ± 21,97 (a)	9,92 ± 0,67 (b)	0,56 ± 0,04 (c)
**Go+Bb+CLIB 1344+Sc**	45,94 ± 5,72 (c,d)	1,04 ± 0,08 (c)	77,89 ± 13,91 (b)	0,32 ± 0,06 (b)	0,90 ± 0,18 (d)	0,84 ± 0,06 (a)	302,44 ± 53,12 (a)	9,78 ± 0,95 (b)	0,55 ± 0,06 (c)

Conditions	Isoleucine: Amyl alcohol	Isoleucine: 2-methylbutyl acetate	Isoleucine: 2-methylbutanoic acid	Threonine: Propanol	Threonine: Propanoic acid	Threonine: Propyl acetate	Threonine: Ethyl propionate
**Sc**	295,36 ± 16,64 (a)	0,50 ± 0,02 (b,c)	1,21 ± 0,09 (a)	24,70 ± 0,42 (b)	1,22 ± 0,06 (b,c)	0,03 ± 0 (d)	0,21 ± 0,02 (a)
**Go+Bb+Sc**	272,16 ± 0,39 (a)	0,23 ± 0,03 (c)	0,81 ± 0,10 (b)	28,46 ± 5,31 (b)	0,94 ± 0,21 (c)	0,05 ± 0,02 (c,d)	0,15 ± 0,02 (b,c,d)
**Go+Bb+CLIB 3741+Sc**	269,17 ± 9,33 (a)	0,71 ± 0,11 (b)	0,22 ± 0,03 (c)	53,43 ± 2,51 (a)	0,94 ± 0,13 (c)	0,12 ± 0,02 (b)	0,16 ± 0,01 (b,c)
**Go+Bb+CLIB 3138+Sc**	248,53 ± 10,65 (a)	1,80 ± 0,10 (a)	0,31 ± 0,04 (c)	51,52 ± 5,25 (a)	1,79 ± 0,04 (a)	0,19 ± 0,03 (a)	0,12 ± 0,01 (d)
**Go+Bb+LMD86+Sc**	298,63 ± 21,97 (a)	1,83 ± 0,12 (a)	0,31 ± 0,02 (c)	47,58 ± 3,81 (a)	1,64 ± 0,17 (a)	0,1 ± 0,01 (b,c)	0,14 ± 0,01 (c,d)
**Go+Bb+CLIB 1344+Sc**	302,44 ± 53,12 (a)	1,72 ± 0,17 (a)	0,28 ± 0,03 (c)	56,40 ± 3,06 (a)	1,58 ± 0,12 (a,b)	0,10 ± 0,03 (b,c)	0,19 ± 0,01 (a,b)

Table S2: Production of volatiles compounds (in mg/L) derived from amino acids, at the end of fermentation by S. *cerevisiae* (Sc) and *M. pulcherrima* strains. The letters (a,b,c,d) are groups according to Tukey test (*p-value* < 0.05).

Conditions	Butanoic acid	Ethyl butanoate	Hexanoic acid	Ethyl hexanoate	Octanoic acid	Ethyl octanoate	Decanoic acid	Ethyl decanoate	Dodecanoic acid	Ethyl dodecanoate
**Sc**	2,38 ± 0,05 (b)	0,49 ± 0,02 (b)	8,70 ± 0,44 (c)	1,54 ± 0,14 (c)	11,70 ± 0,95 (c)	1,94 ± 0,18 (b)	4,26 ± 0,32 (b)	0,77 ± 0,09 (a)	0,21 ± 0,02 (b,c)	0,07 ± 0,01 (a)
**Go+Bb+Sc**	2,39 ± 0,13 (b)	0,60 ± 0,05 (b)	10,13 ± 0,70 (b,c)	1,84 ± 0,15 (b,c)	10,05 ± 2,97 (c)	2,26 ± 0,11 (a,b)	3,16 ± 0,59 (c)	0,84 ± 0,34 (a)	0,20 ± 0,01 (b,c)	0,09 ± 0,03 (a)
**Go+Bb+CLIB 3741+Sc**	2,31 ± 0,25 (b)	0,85 ± 0,03 (a)	10,19 ± 0,97 (b,c)	2,07 ± 0,12 (b)	8,51 ± 0,61 (c)	2,45 ± 0,34 (a,b)	2,07 ± 0,38 (d)	0,94 ± 0,09 (a)	0,17 ± 0,01 (c)	0,08 ± 0 (a)
**Go+Bb+CLIB 3138+Sc**	3,4 ± 0,30 (a)	0,90 ± 0,04 (a)	16,19 ± 0,77 (a)	2,40 ± 0,13 (a)	19,41 ± 1,06 (a)	2,63 ± 0,13 (a)	5,56 ± 0,25 (a)	0,83 ± 0,03 (a)	0,30 ± 0,01 (a)	0,09 ± 0,01 (a)
**Go+Bb+LMD86+Sc**	2,57 ± 0,27 (b)	0,84 ± 0,03 (a)	11,54 ± 1,42 (b)	2,04 ± 0,12 (b)	16,31 ± 1,47 (a,b)	2,21 ± 0,09 (a,b)	5,13 ± 0,19 (a,b)	0,74 ± 0,04 (a)	0,31 ± 0,04 (a)	0,08 ± 0,01 (a)
**Go+Bb+CLIB 1344+Sc**	2,15 ± 0,05 (b)	0,84 ± 0,06 (a)	9,25 ± 0,88 (b,c)	2,03 ± 0,17 (b)	15,34 ± 0,51 (b)	2,06 ± 0,24 (a,b)	5,01 ± 0,30 (a,b)	0,70 ± 0,13 (a)	0,25 ± 0,01 (b)	0,06 ± 0 (a)

Table S3: Production of volatiles compounds (in mg/L) derived from medium-chain fatty acids, at the end of fermentation by S*. cerevisiae* (Sc) and *M. pulcherrima* strains. The letters (a,b,c,d) are groups according to Tukey test (*p-value* < 0.05).

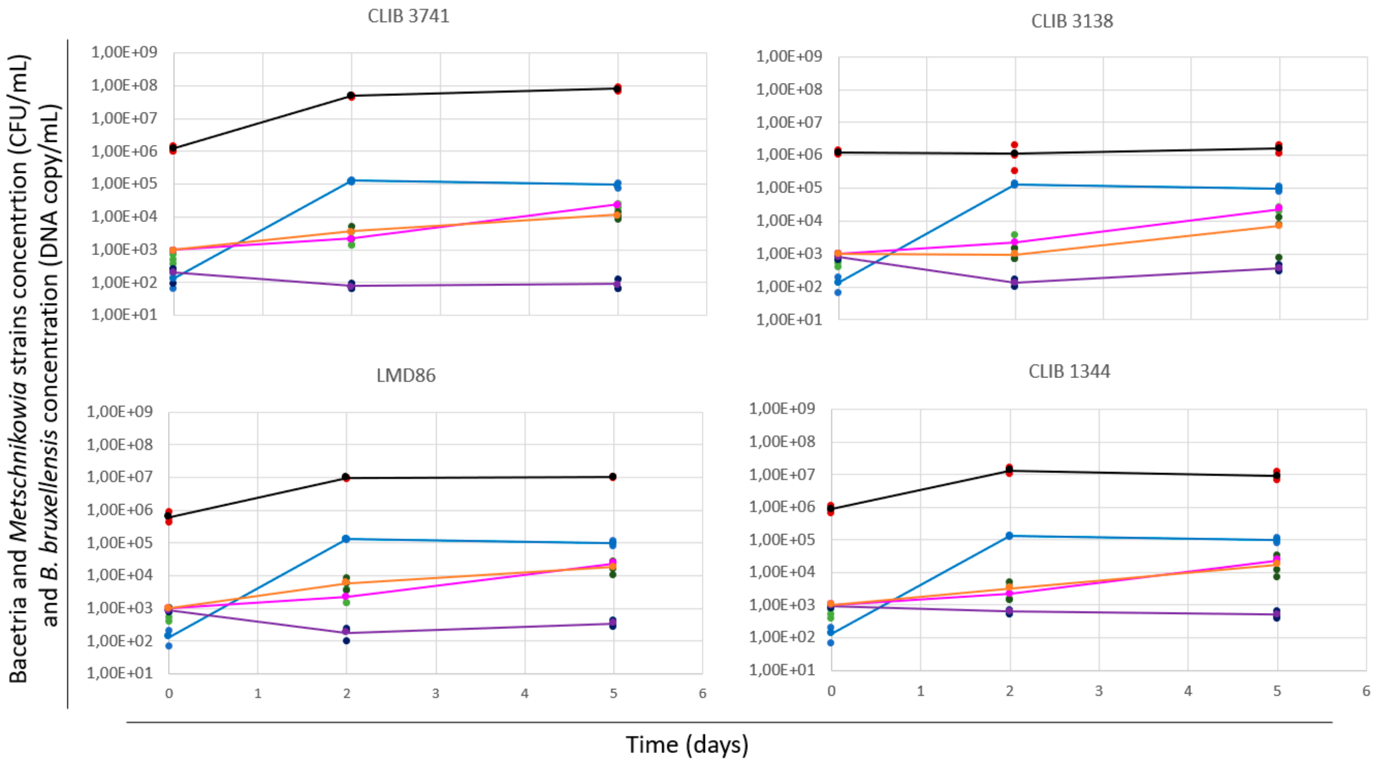

Figure S4: Growth follow at day 2 and day 5, of the spoilage microorganisms *G. oxydans* and *B. bruxellensis* and *M. pulcherrima* strains in Chardonnay must. The light blue dots represent the growth of *G. oxydans* in co-culture with *B. bruxellensis* and *S. cerevisiae* and the blue line the average ; the darker blue dots represent the growth of *G. oxydans* in co-culture with *B. bruxellensis*, *S. cerevisiae* and *M. pulcherrima* strains and the purple line the average ; the light green dots represent the growth of *B. bruxellensis* in co-culture with *G. oxydans* and *S. cerevisiae* and the pink line the average ; the darker green dots represent the growth of *B. bruxellensis* in co-culture with *G. oxydans*, *S. cerevisiae* and *M. pulcherrima* strains and the orange line the average ; the red dots represent the growth of *M. pulcherrima* strains in co-culture with *G. oxydans*, *B. bruxellensis* and *S. cerevisiae* and the black line the average.
